# Transitioning from epicutaneous to oral peanut immunotherapy

**DOI:** 10.3389/falgy.2023.1089308

**Published:** 2023-02-06

**Authors:** Lauren Wong, Laurie Kost, Brent Anderson, Andrew Long, Sayantani B. Sindher, R. Sharon Chinthrajah, William J. Collins

**Affiliations:** ^1^ Sean N Parker Center for Allergy and Asthma Research at Stanford University, Stanford University, Stanford, CA, United States; ^2^ Division of Hospital Medicine, Department of Medicine, Stanford University School of Medicine, Stanford, CA, United States

**Keywords:** peanut allergy, epicutaneous immunotherapy (EPIT), oral immunotherapy (OIT), peanut patch, desensitization

## Abstract

**Introduction:**

Epicutaneous immunotherapy (EPIT) has been tested in clinical trials for children with peanut allergy (PA) for its safety and efficacy in inducing desensitization. Aside from peanut avoidance and symptom management, oral immunotherapy (OIT) is another option for PA patients. However, OIT can be associated with adverse events and pose safety concerns to children and their caregivers.

**Methods:**

This study assessed 27 children who successfully completed a peanut EPIT trial. 18 of them transitioned to peanut OIT with starting doses ranging from 10–600 mg of peanut protein. Our aim was to learn more about the EPIT to OIT experience through descriptive survey responses and to gather information that may support the sequential use of the two immunotherapies for safe and positive outcomes that may not be achieved by either alone.

**Results:**

Overall, children and their caregivers had less anxiety about starting OIT after having had peanut exposure through EPIT. Most children who transitioned from EPIT to OIT had no or minor symptoms initially, with symptoms lessening later in OIT. Most were also able to maintain or increase their peanut dose over time, achieving maintenance doses of 60–2,000 mg.

**Discussion:**

In comparison with current literature on OIT for PA in children, the reported symptoms appeared less severe and less prevalent in the EPIT to OIT group. However, there were 3 participants who withdrew from OIT due to the development of intolerable symptoms. This study provides initial data in support of EPIT to OIT, and larger randomized controlled trials assessing effectiveness of the two therapies together are warranted.

## 1. Introduction

Peanut allergy (PA) is one of the most common food allergies in Western countries and currently affects approximately 2% of the general population and up to 8% of children in the United States (US) (1, 2). It is typically diagnosed in early childhood and can be a serious and potentially life-threatening condition that poses a burden on the quality of life of children and their families. Those with PA experience higher rates of accidental exposure, severe reactions, and anaphylaxis than other food allergies (1). Current options for the management of PA include peanut oral immunotherapy (OIT - either with an FDA-approved product now available in the United States or through private practice), sublingual immunotherapy (SLIT), or strict avoidance of peanuts and use of epinephrine in the case of accidental exposure (3, 4).

Immunotherapy is an important treatment option to reduce reactivity to an allergen *via* the desensitization process. In OIT, an individual ingests set amounts of allergen and builds up to a maintenance dose, which then may be continued or lowered for long-term dosing to maintain desensitization (2). However, for many peanut allergic individuals and their families, aiming to achieve peanut desensitization through OIT is anxiety-inducing and raises concerns of systemic reactions (5). EPIT is a relatively new therapy that is being tested for individuals with PA, in which a specific quantity of peanut is delivered on the upper layers of the skin (6). The aim of EPIT is to provide a safer way to desensitize those with PA by exposing them to peanut without direct contact between peanut antigen and the gastrointestinal tract to avoid a more dangerous systemic reaction.

**Figure 1:**
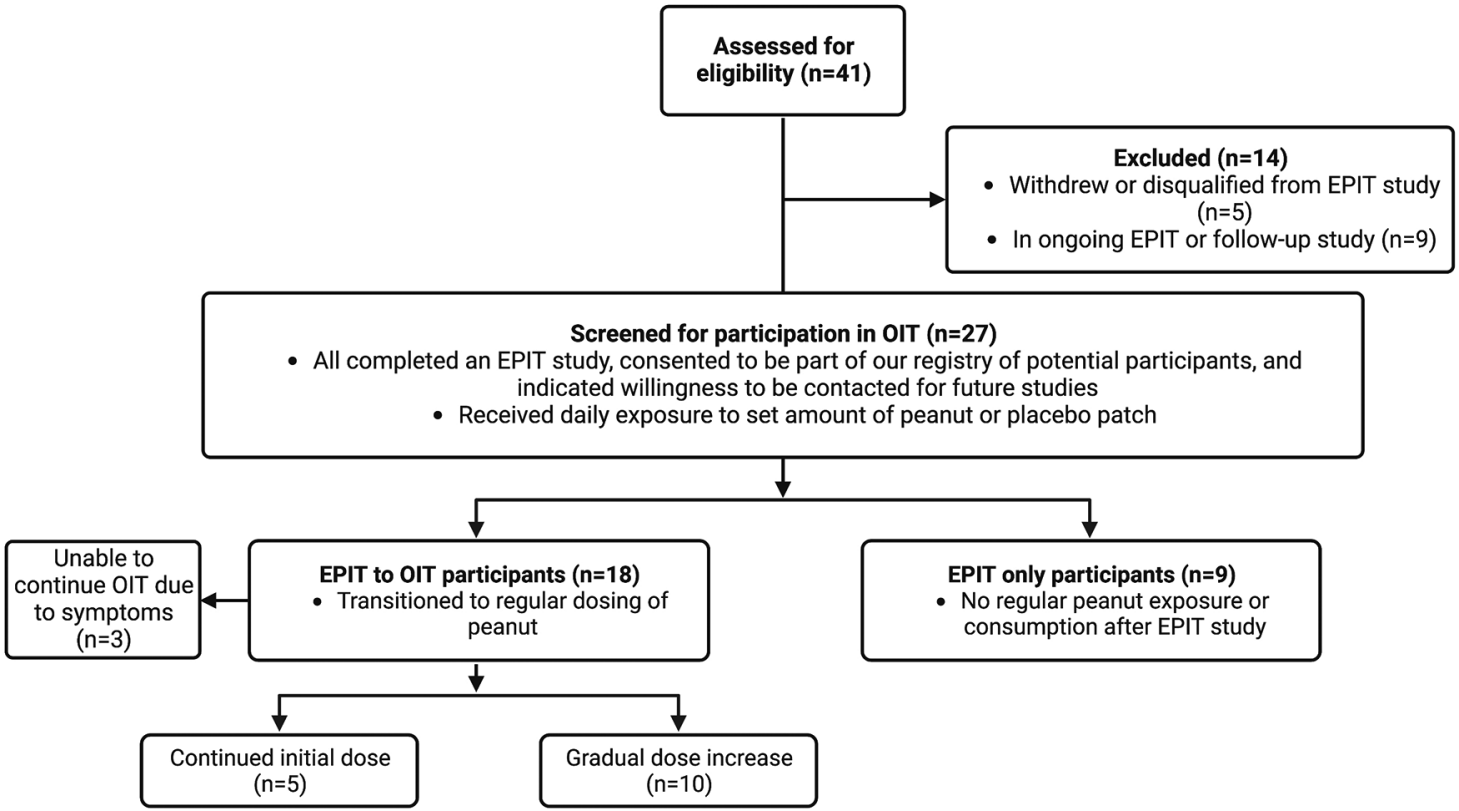
Participant progression after completion of EPIT study.

Clinical trials to test the safety and efficacy of EPIT for the treatment of PA in children have shown significant treatment response characterized by the ability to tolerate consumption of a predefined dose of peanut protein compared to placebo with better response at higher patch doses and in younger aged children (6–10). The majority of adverse reactions are local patch site reactions. Although safety and compliance may be improved with EPIT, the peanut doses administered through the patch alone cannot achieve the same degree of desensitization compared to OIT. The aim of this observational study was to assess responses of children who completed an EPIT study and have since either transitioned to oral peanut consumption including OIT or chose not to continue with peanut dosing (Figure 1). We were interested in investigating the combination therapy of EPIT and OIT–specifically whether EPIT can help prepare an individual for OIT and mitigate reactions experienced during OIT.

## 2. Materials and methods

Participants of any age who had completed an EPIT study (NCT02636699, NCT03013517, NCT02916446, NCT03211247, NCT03859700, NCT01675882, NCT01955109) at our center, consented to be part of our registry of potential participants, and indicated willingness to be contacted for future studies were considered eligible. Study materials were approved by our local IRB. Caregivers were contacted over a period of 4 months to ask to complete an electronic survey *via* REDCap. Surveys included questions to confirm EPIT study participation and knowledge of their child's experimental group. Other questions included age at the start of the study, if they initiated OIT, and details of the transition, dosing, and symptoms for those who did. Questions were either multiple choice or open-ended short answers. The survey also asked if participants were still eating peanut, and at what amount, frequency, and method of intake. Space was given for additional comments about the experience with the peanut patch and OIT.

Data was compiled and responses were grouped into EPIT to OIT and EPIT only. Follow-up interviews were done for the EPIT to OIT group, and they were able to provide most recent serum Immunoglobulin E (IgE) and skin prick test (SPT) results for peanut or were given the option of repeating these tests in our clinic. Phone interviews to the EPIT only group were done to elicit more detailed descriptions of why families decided not to proceed with OIT.

## 3. Results

### 3.1. EPIT to OIT group

**Figure 2:**
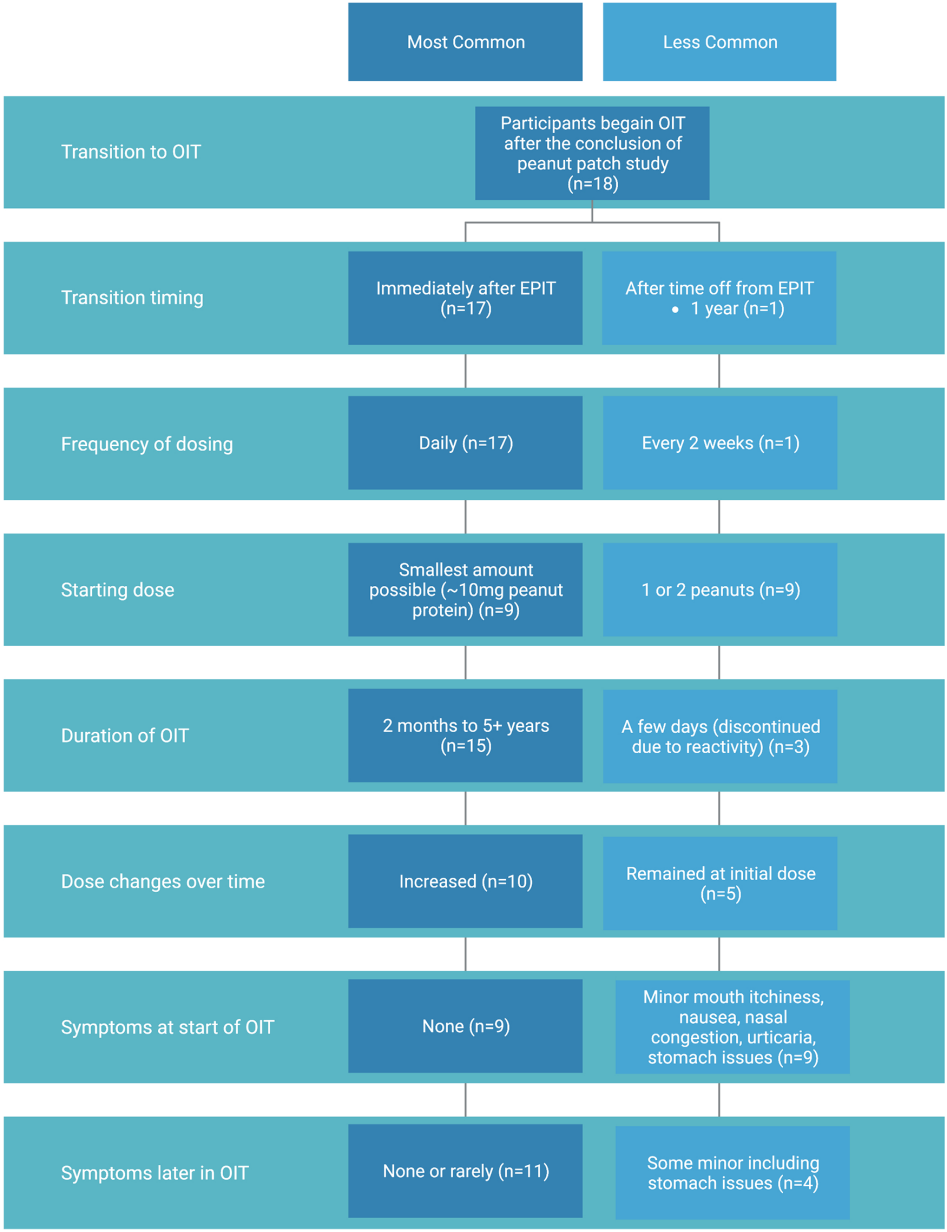
Summary of OIT experience of participants who transitioned from peanut patch to OIT including most common (dark blue) and less common (light blue) methods and outcomes.

We received a total of 27 survey responses, 18 of which were in the EPIT to OIT group (Figure 1). Ages of these participants ranged from 2–11 years old at the start of their peanut patch study, with current ages of 4–18 years old. All but one of these participants had been in the treatment group of their EPIT studies and received a dose of either 100 or 250 µg of peanut epicutaneously. The majority (15/18) transitioned to OIT soon after their EPIT study without further adjunctive therapy, while 2 participants transitioned to OIT alongside dosing with omalizumab (including the one participant who was not in the experimental group of the patch study). One participant waited for a year after EPIT to start OIT (Figure 2). OIT participants transitioned with the help of a physician, so it is assumed that the initial dose was determined by their allergist, although most were starting at the smallest dose possible, presumably due to potential risk for adverse events. Participants did not specify whether their OIT was done with FDA-approved peanut powder, but peanut intake was reported to be whole or portions of peanut or peanut flour as recommended by local allergists. Dose escalation varied, with some choosing to stay at the same initial dose and others escalating if no serious adverse effects were observed. There was no standardized dose escalation, as these individuals underwent OIT with different providers after completion of their EPIT study. All children in this group were dosing OIT daily with the exception of the individual who started OIT a year after finishing the patch study. That individual was consuming peanut doses every two weeks.

Our survey and follow-up interviews elicited descriptive responses from caregivers about children's symptoms with the initial start of OIT and ongoing dosing. At the start of the OIT transition after EPIT, 50% of participants in our cohort did not experience any symptoms. Those who did experience symptoms reported minor nausea, congestion or itchiness of the mouth (6/18), stomach issues (2/18), and urticaria (1/18). Overall, most had no or minor symptoms that only lasted for the first few days of OIT. When asked about symptom presentation later in OIT dosing, a majority of participants had none or rarely any (11/18). Of those with some symptoms, one individual had continued stomach issues and had to move slowly through updosing but was still able to continue increasing to reach a maintenance peanut dose.

Three participants, all of whom were in an experimental group of a patch study, experienced symptoms that led to a decision to terminate OIT. One of them who started with no symptoms began reacting on day 9 of a daily low dose (<10 mg of peanut protein) and stopped due to abdominal pain and urticaria. Another participant, who started at a dose of one third of a peanut, developed eosinophilic esophagitis after OIT and stopped eating peanut to resolve the condition. The third participant did notice improvement with the peanut patch and admitted to feeling more comfortable with peanut exposure but reacted a few days into OIT and chose to stop. This participant suggested that perhaps their starting dose was too high and may have had a more positive experience at a lower initial dose.

There was variation in how long participants had been treated with OIT, ranging from months to years. The starting dose also varied with the range of doses including 10 mg, ¼ of a peanut (∼75 mg), 1 peanut (∼300 mg), and 2 peanuts (∼600 mg). Of those who tolerated the start of OIT (15/18), a majority (10/18) were able to increase their dose over time and are now eating peanut daily. The remaining participants did not increase their OIT dose over time but continued with peanut consumption (5/18). Two of those families are using OIT as a method of developing protection against accidental exposure for their child and did not have interest in increasing the dose to incorporate peanut in their everyday diet. Another has not followed up with their allergist about a dose increase. Not all families initiated OIT with the aim of reaching daily or unlimited peanut consumption, thus not all participants had a target peanut dose. Overall, most who transitioned to OIT were able to stay at or increase their dose of peanut over time, with final maintenance doses ranging from 60–2,000 mg as determined by participant and/or family preference and the advice of their allergist.

#### 3.1.1. Descriptive analysis

Descriptive responses were collected about family experiences with EPIT and OIT. Of the positive comments, caregivers shared how a successful oral food challenge without significant dose limiting symptoms after the patch study gave them confidence that they could start OIT without concern. The EPIT to OIT experience also eased caregiver concern about their child accidentally eating peanuts or coming into contact with peanut in a school environment. Two children who were on omalizumab while starting OIT had a smooth transition. One caregiver commented that it was life-changing and being on the patch and OIT helped their child go from high sensitivity to specks of peanut in the air to tolerating whole peanuts after 6 months.

#### 3.1.2. Clinical testing

Of the participants from whom we were able to collect skin prick and serum IgE test results after having been on OIT after the peanut patch, all continued to have a positive SPT and/or elevated IgE (Table 1).

**Table 1:** Skin prick test (SPT) and serum immunoglobulin E (IgE) results from EPIT to OIT participants.

Participant #	SPT peanut average wheal (mm)	Peanut IgE
1	8	Unable to collect
2	5.5	9
3	4	6.03
4	Unavailable	>100
5	Unavailable	7.02
6	11	ara h2: 21.90
7	5	24.6
8	8	>100
9	6	3.97
10	6	3.97
11	7	>100
12	6.5	4.93

### 3.2. EPIT only group

**Figure 3:**
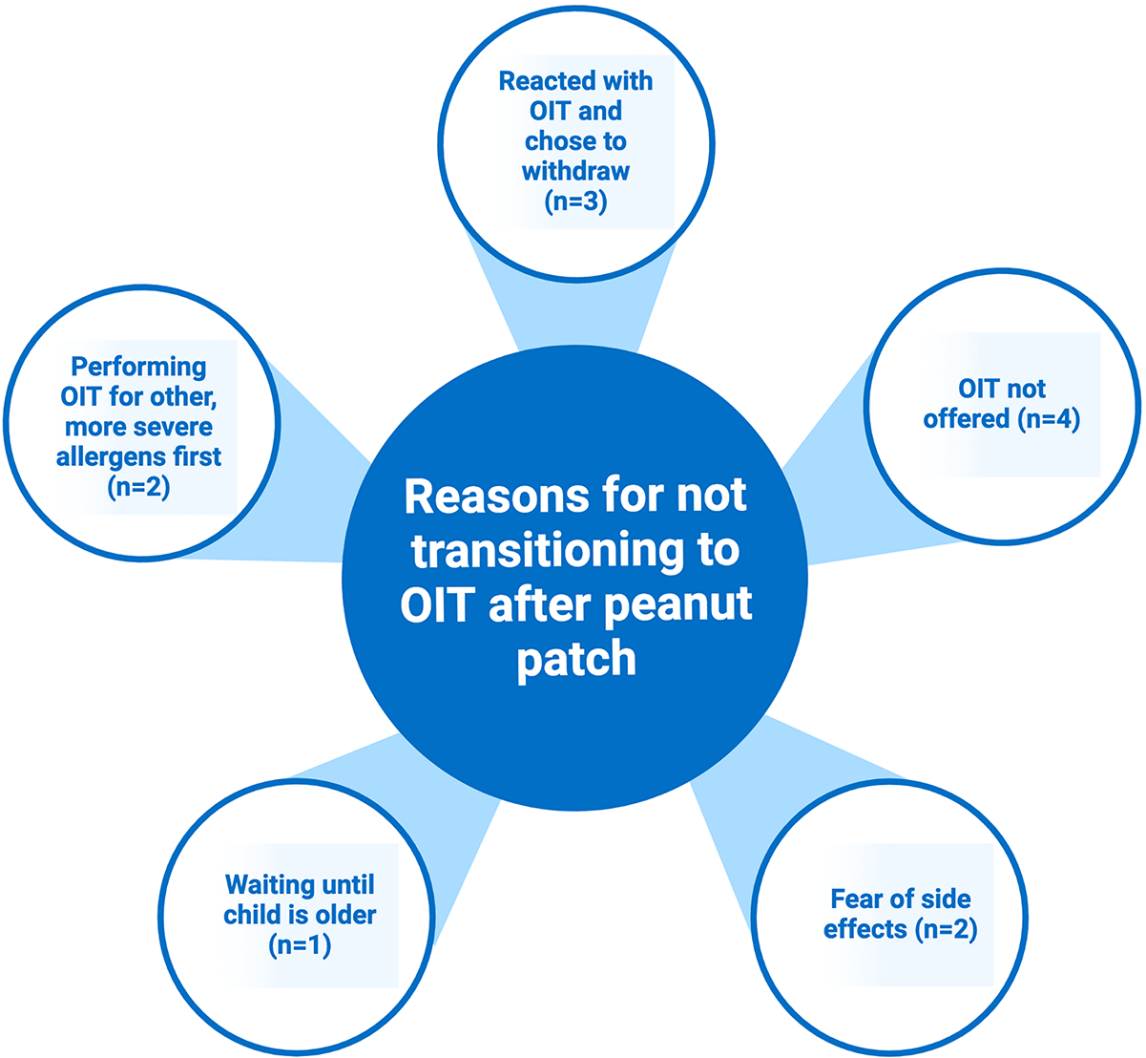
Reasons families chose not to transition to OIT after peanut EPIT.

Those in the EPIT only group were asked about why they did not transition to OIT (Figure 3). A common reason was simply that they were not offered an option to continue to OIT after the study, and there was a lack of follow up with their allergist. When asked if they would consider OIT in the future, these caregivers all responded yes. Other caregivers worried about the possibility of a reaction with OIT and feared that OIT would have more symptoms than those already experienced with the patch. Some wanted to wait until their child was older to leave the decision of desensitization to them. One family chose to do OIT for more severe allergens first before proceeding with peanut.

## 4. Discussion

This observational study evaluated the experience of peanut allergic children who participated in an EPIT study who did and did not transition to oral peanut consumption with OIT. In total, 27 respondents who previously completed peanut patch studies were surveyed with 18 of them having attempted OIT after EPIT. Although there was variation in dosing, our group focused on the experience of those who transitioned to OIT, particularly what type of symptoms they had and if they changed over time with or without dose escalation. Two participants were put on higher starting doses due to their concurrent use of omalizumab. Starting doses of other participants were likely selected based on participants' food challenge results at the end of EPIT study and provider discretion. Our results indicate potential reduction in symptoms and improvement in quality of life with completion of EPIT prior to start of OIT. More specifically, the majority of participants had no or minor symptoms at the start of OIT that persisted for no more than a few days. Common symptoms included itchiness and congestion. Later in OIT, symptoms improved for most and OIT was continued at the same or increased dose. However, 3 participants in our cohort were not able to tolerate OIT, which is a relatively high rate of dropout, though it may potentially be inflated by our low sample size. An alternative explanation for this group of participants may be their peanut allergy was too severe such that they would not have tolerated OIT with or without EPIT. Instead, it may be specific only for individuals with the potential to tolerate OIT that preceding EPIT improves their experience with OIT. With regards to quality-of-life measures, children and caregivers reporting having less anxiety before and during OIT because participants had had prior peanut exposure with EPIT. The risk of peanut contamination and accidental ingestion was not as worrisome for families after having successfully gone through EPIT and OIT.

Studies of immunotherapy for peanut allergy generally only explore one method of immunotherapy and vary in the parameters and methods used for symptom reporting. However, in comparing the types of symptoms and proportion of participants affected to our survey responses, there may be benefit in initiating EPIT prior to OIT to lessen the severity of reactions experienced in the desensitization process. Jones et al. performed peanut OIT on subjects aged 1 to 16 years old, with a majority starting at the same or slightly higher dose than the lowest dose of 10 mg used by our participants and increasing up to 1,800 mg daily (11). While a relatively low percentage of reactions occurred per dose (3.7% of 14,773 doses), all participants experienced at least one minor symptom with buildup dosing, whereas only 50% of participants in our cohort reported symptoms at the start of OIT and 61% reported no or rare symptoms thereafter. Symptoms were similarly most common in the upper respiratory tract and skin.

In a separate study by Varshney et al., peanut OIT began at lower doses of 1.5–12 mg but subjects could increase up to a 4,000 mg maintenance dose (12). In this study, reactions occurred with 1.2% of 407 build up doses, which is potentially more comparable to reported symptoms from those in our EPIT to OIT group. It should be noted that the retrospective nature of our study may lead to underreporting of symptoms by participants due to incomplete recall. However, the potential that subjects may have symptoms mitigated by undergoing EPIT before the start of OIT is deserving of further study.

Importantly, our EPIT to OIT subjects did not undergo systematic initial dose escalation on the first day of OIT unlike those in most OIT studies. Given that symptom frequency is often high during initial dose escalation (IDE) days in OIT studies, the ability to start at a potentially higher dose and experience low rates of symptoms with preceding EPIT therapy presents another potential advantage. For example, ninety-two percent of participants in the Jones et al. study experienced symptoms during the IDE with 4 requiring treatment with epinephrine (11). In the study by Varshney et al., forty-seven percent of subjects required anti-histamine treatment for symptoms and 2 required epinephrine (12). Two participants did not reach a cumulative dose of at least 1.5 mg and were considered treatment failures. Although our survey participants were not specifically questioned about antihistamine and epinephrine use, there were no reports of severe symptoms requiring these medications. Overall, the symptoms described for the initial phase of this study seemed to be less severe with EPIT to OIT participants, despite most having a higher starting dose.

The IMPACT trial studied children 1–3 years old with OIT up to a maintenance dose of 2,000 mg peanut protein per day (13). 98% of participants had at least one dosing reaction, which were predominantly categorized as mild to moderate. Of the 96 experiencing moderate symptoms, 21 were treated with epinephrine during updosing or maintenance phases. A direct comparison to our survey group is difficult due to the higher maintenance doses that were achieved in this study. Our percentage of early and total withdrawal was 16.7%, while the IMPACT trial had 15.6% of participants withdraw before 134 weeks. However, a total 27% of IMPACT participants withdrew from the OIT group, which brings up a consideration of patient safety and the potential for longer term feasibility and the avoidance of severe reactions with EPIT preceding OIT.

The phase 3 PALISADE study included a total of 496 participants ages 4–17 years old (14). Their median maximum tolerated initial dose was 10 mg, which was the lowest of the starting dose range amongst the EPIT to OIT cohort. Over 50% of children in the study experienced more than one adverse event (AE) in the initial dose escalation, with the main ones being abdominal pain, oral pruritus, nausea, and throat irritation. EPIT to OIT participants did not experience as wide of a range of symptoms as seen in PALISADE; however, this may be more a reflection of our small sample size. Notably, only 5.6% of our EPIT to OIT group experienced more than one AE at the start of OIT. The proportion of participants experiencing more than one AE in the PALISADE study remained high throughout OIT, with more than 95% of participants aged 4–17 having events during the updosing period and 87% during the maintenance phase. In contrast, the percentage of EPIT to OIT children exhibiting AEs was 50% at maximum and went down to 40% as peanut dosing went on. The lower percentage of severe and systemic reactions with the progression of OIT could suggest that EPIT can play a protective role in avoiding these types of reactions in OIT.

In the POISED study the participant group was aged 7–55 years (15). Despite the greater age range, in evaluating the types of symptoms reported, the most common were mild gastrointestinal (in 83%) and skin disorders (in 43%). At the beginning of OIT, almost all of the participants in POISED experimental groups (91% and 95%) experienced AEs. The AEs did decrease over time, as with the EPIT to OIT cohort. Overall, a much lower proportion of participants in the EPIT to OIT group displayed gastrointestinal (11%) and skin reactions (5.6%) than in POISED, with a smaller amount experiencing symptoms in general through OIT. However, it should be noted that the maximum dose reached in this study was 4 grams.

Given participants in our cohort were not under a formal protocol for OIT, it may be more appropriate to compare outcomes to those observed from peanut OIT done in actual clinical practice. In one retrospective review of real-world peanut OIT from North Texas, 79% of patients who began dose escalation were able to reach a target dose of 2,000–3,000 mg; however, 37% experienced primarily gastrointestinal (GI) side effects and 13.7% experience persisting GI symptoms including vomiting more that 2 hours after dosing (16). Similarly, 83% of participants in our cohort were able to continue OIT after starting, though with a lower range of target dosing. As before, symptoms in our cohort appeared to be less frequent, though this may at least in part be due to lower final doses. However, in another study of the OIT experience of a large private practice in New England, at least one GI symptom occurred in 84% of patients during build up though 89% progressed through build up to maintenance dosing (17).

The comparison of EPIT to OIT with studies of OIT alone are certainly interesting, but they must be made with caution. While there was a range of starting and maintenance doses for the EPIT to OIT participants, many OIT studies went to higher maintenance doses. Although there are likely variations between how symptoms are measured, reported, and graded, the general types of reactions and proportions of participants experiencing them suggests that EPIT to OIT may offer a safer approach to desensitization of PA. A study using quantitative risk assessment for the risk of an allergic reaction with and without EPIT for PA also provides support that treatment with EPIT can help prevent a moderate/severe allergic reaction (18).

Of note, SPT and IgE testing for peanut remained positive in all participants for which it was available after EPIT to OIT, though the significance of this is uncertain. Without pre-EPIT baseline values for the SPT and serum IgE levels, it is difficult to interpret the lab results, and previous studies do not necessarily show a reduction in these markers after OIT alone (19–21). Further prospective research should include such testing to determine if there is a meaningful change in these markers that could warrant their use in the interpretation of EPIT and OIT responses.

Since our study was strictly observational, we relied on recall and self-reporting from families, which could have inaccuracies. Although there are a limited number of participants who have successfully completed a peanut patch study and transitioned to OIT, we would have liked to gather data from more participants. It would have also been useful to have access to initial IgE and SPT results from patch studies to act as a point of comparison to the most recent tests from EPIT to OIT participants.

In the future, larger trials of peanut EPIT and EPIT to OIT would allow for more information to be gathered about its efficacy and safety. Having a placebo-controlled trial to allow comparison of EPIT only, OIT only and EPIT to OIT groups on similar dosing regimens would enable better comparison between the therapies. Testing whether participants from different groups are able to reach the same maintenance and exit food challenge dose, whether dosing symptoms may be mitigated with EPIT to OIT, and if EPIT to OIT enables a higher final peanut dose could provide potential support for this combination therapy. Another area for further study is age difference–it would be interesting to involve multiple age groups to assess if age affects the outcome of EPIT to OIT. Studies could also weigh the use of multiple immunotherapies in different combinations such as by studying the use of SLIT prior to OIT compared to the use of EPIT prior to OIT.

PA is a prevalent and potentially life-threatening condition with only one currently approved therapy. EPIT has been tested in PA clinical trials, and here we evaluated the experience of children who transitioned from EPIT to OIT. Though there is still more research that needs to be done, our survey of EPIT to OIT participants and comparison of those results to OIT literature suggests that this therapeutic approach could provide a safer method of targeting PA than either immunotherapy alone.

## Data Availability

The raw data supporting the conclusions of this article will be made available by the authors, without undue reservation.
